# Chromatin state dynamics during NK cell activation

**DOI:** 10.18632/oncotarget.16688

**Published:** 2017-03-29

**Authors:** Yang Li, Jin Wang, Jie Yin, Xinhua Liu, Minghang Yu, Ting Li, Han Yan, Xi Wang

**Affiliations:** ^1^ Department of Cell Biology, 2011 Collaborative Innovation Center of Tianjin for Medical Epigenetics, Laboratory of Epigenetics in Development and Tumorigenesis, Tianjin Research Center of Basic Medical Sciences, Tianjin Key Laboratory of Medical Epigenetics, Tianjin Medical University, Tianjin, China; ^2^ Department of Biochemistry and Molecular Biology, 2011 Collaborative Innovation Center of Tianjin for Medical Epigenetics, Tianjin Key Laboratory of Medical Epigenetics, School of Basic Medical Sciences, Tianjin Medical University, Tianjin, China

**Keywords:** NK cell, activation, histone modification, poised state, small molecule inhibitor, Immunology and Microbiology Section, Immune response, Immunity

## Abstract

Studies of Natural Killer (NK) cell cytotoxicity have mainly focused on the balance of activating and inhibitory receptors, signaling transduction, calcium influx, formation of immune synapse, and cytolytic degranulation. However, little is known about the chromatin state of NK cells and the impact of its changes during target recognition. In this study, we investigate the contribution of chromatin state dynamics during NK cell activation by comprehensively analyzing a set of microarray data and two sets of Chromatin Immunoprecipitation-Sequencing (ChIP-seq) data. We find that the expression of several histone demethylases and methyltransferases was influenced upon stimulation. Furthermore, we notice that a series of genes, including PI3KCA, NFATC1and TNFSF9, which play important roles during NK cell activation, were at ‘poised’ state prior to activation, and that modifications of H3K4me3 and H3K27me3 on these promotors were sensitive to stimulation with Phorbol Myristate Acetate (PMA) and Ionomycin (Iono) in the NK92MI cell line. Finally, we demonstrate that a series of small molecule inhibitors, which are specific to H3K4 and H3K27 modification, enhance degranulation or the expression levels of IFN-γ and TNF-α. Our results suggest that the histone modification state has a profound impact on NK cell activation, and provide novel insights into the regulation of NK cellular cytotoxicity and immunoregulatory function by chromatin state dynamics.

## INTRODUCTION

NK cells were originally considered pivotal players in the vertebrate immune system that could rapidly recognize and eliminate transformed or virus-infected cells without the requirement of previous immune sensitization. They were later recognized as a specific, cytotoxic lymphocyte lineage endowed with immunoregulatory activity, producing proinflammatory chemokines and cytokines [1]. Upon target recognition, NK cells obtain the activating signal to enter a well-defined, multistep process starting with extracellular Ca^2+^ fluxing into the cytoplasm, cytoskeleton remodeling, and a cell-cell specialized area formation called the cytolytic immune synapse. Then the cytotoxic granules together with the microtubule organizing center (MTOC) migrate toward the immune synapse. At the same time, the resulting boost in calcium signaling generates universal and sufficient cytoplasmic Ca^2+^ sensor calmodulin (CaM) binding to various enzymes or channels, modulating their activity. Phosphatase calcineurin is one of the targets of CaM, which dephosphorylates NFAT and induces its nuclear translocation, thereby mobilizing NFAT-dependent transcription, including cytokines and chemokines [2]. Finally, effectors (cytokines and chemokines) and granules (granzymes and perforin) are trafficked and secreted to perform their functions [3]. In this way, resting NK cells turning into an entirely different state (activated NK cells) that is characterized by their ability to kill and secrete effector cytokines during target recognition.

NK cellular cytotoxicity has been an ongoing conundrum ever since the activity was first described in 1975 by Herberman, who observed that mouse lymphoid cells naturally killed syngeneic acid allogeneic tumors. Since then, major advances of NK cell cytotoxic activity and cytokine production have focused on the balance of activating and inhibitory receptors, signaling pathways, calcium influx, formation of immune synapse, and cytolytic degranulation. Yet crucial questions remain regarding the chromatin state of NK cells during target recognition.

In eukaryocyte, the chromatin state is considered to contribute to the regulation of gene expression. The post-translational modifications (PTMs) of histone include, but are not limited to, histone phosphorylation, acetylation, ubiquitylation, and methylation, all of which are thought to regulate gene expression by recruiting key regulators or by manipulating chromatin architecture. These modifications have the capacity to influence biological processes in the context of development and cellular responses to environmental cues. Therefore, a delicate balance between stability and dynamics in histone PTMs is imperative for specific gene expression.

Here, we start by comprehensively analyzing a set of microarray data and two sets of ChIP-seq data, to address whether a series of genes exhibiting a ‘poised’ state may play an important role during NK cell activation. Then we conduct intervening studies using several small-molecule compounds to change the chromatin state of NK cells, demonstrating that the UNC1999 inhibitor could enhance the degranulation of NK92MI, while both the MM-102 and OG-L002 inhibitors could influence the expression of IFN-γ and TNF-α. The results provide new insights into the regulation of NK cells cytotoxicity and immunoregulatory function.

## RESULTS

### Gene expression profiling reveals NK cells undergo a dramatic transformation during activation

**Figure 1:**
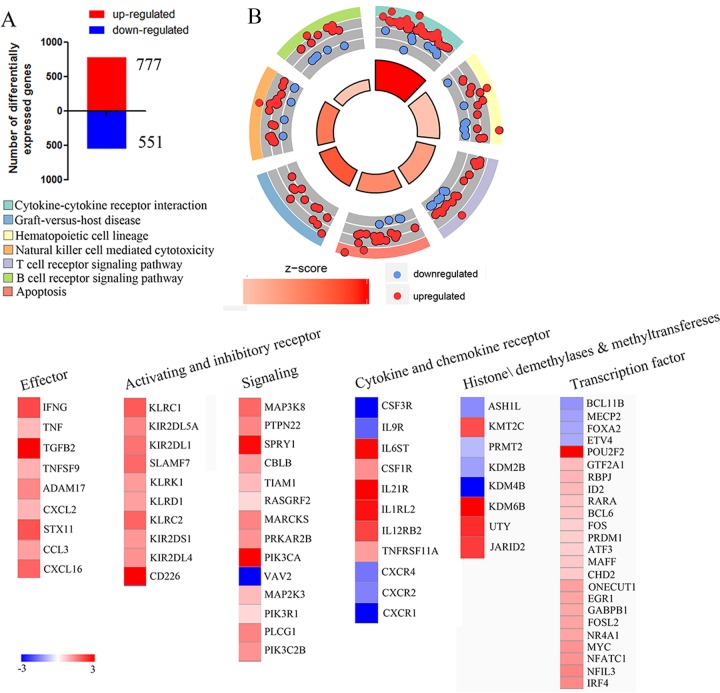
Gene expression profiling reveals NK cells undergo a dramatic transformation during activation **A**. Microarray analysis revealed that 777 genes were upregulated (red) and 551 genes were downregulated (blue). **B**. Circle plot; the inner ring is a bar plot where the bar height indicates significance, and color corresponds to the z-score. Every color of the outer ring corresponds to a different, representative signaling pathway, and scatterplots display expression levels (logFC) for the genes. **C**. Heat map of cell surface receptors, signaling components, transcription factors, methyltransferase and demethylase as well as genes associated with NK cells effector that were significantly differentially expressed by comparing activating NK cell *versus* resting NK cell are plotted.

Gene expression profiling has provided great insight into NK-cell function [8–10]. NK cells respond rapidly to target cells, and we supposed that analyzing the changes in gene expression that occurred during the early stages of NK cells activation by target cells might uncover new features of these responses. Gene expression profiling of resting-NK and activated-NK fractions was performed as described [11] and limma package of R software was used to analyze the Differential Expression Genes (DEGs). We set the thresholds for |log FC| > 1 and a *P* value < 0.05, to identify 777 up-regulated and 551 down-regulated genes (Figure 1A). Then we uploaded the whole set of DEGs to the Database for Annotation, Visualization and Integrated Discovery (DAVID) database to identify the Kyoto Encyclopedia of Genes and Genomes (KEGG) signaling pathways. A summary of our KEGG results is given in Figure 1B. In detail, most of the enriched signaling pathways were highly correlated with immune responses pathways involving cytokine-cytokine receptor interaction, Natural killer cell mediated cytotoxicity and T/B cell receptor signaling. Genes associated with hematopoietic cell lineage, apoptosis as well as an overwhelming majority of the clustered related genes were up-regulated (Figure 1B). A similar result was obtained by unbiased Gene Set Enrichment Analysis (GSEA) analysis (Supplementary Figure 1).

We then focused on genes associated with the immune activation phenotype. Several genes encoding cell surface receptors, signaling components, transcription factors, as well as genes associated with NK cell effector function were identified in our data, and most of them were upregulated (Figure 1C). Furthermore, to investigate the contribution of methyltransferase and demethylase on regulating the cytotoxic activity and cytokine production of NK cells, we performed an assay to identify all differentially expressed histone methyltransferases and demethylases genes upon activation of human NK cells. The results show that eight methyltransferase and demethylase genes exhibit altered expression during the target cell-recognition stage (Figure 1C). Thus, this data suggests that NK cells experience a dramatic transformation during the recognition phase, and the chromatin-modifying enzyme may play critical roles in NK cell activation.

### Gene expression of histone methytransferases and demethylases screened from microarray results were verified by qPCR and western blot

**Figure 2:**
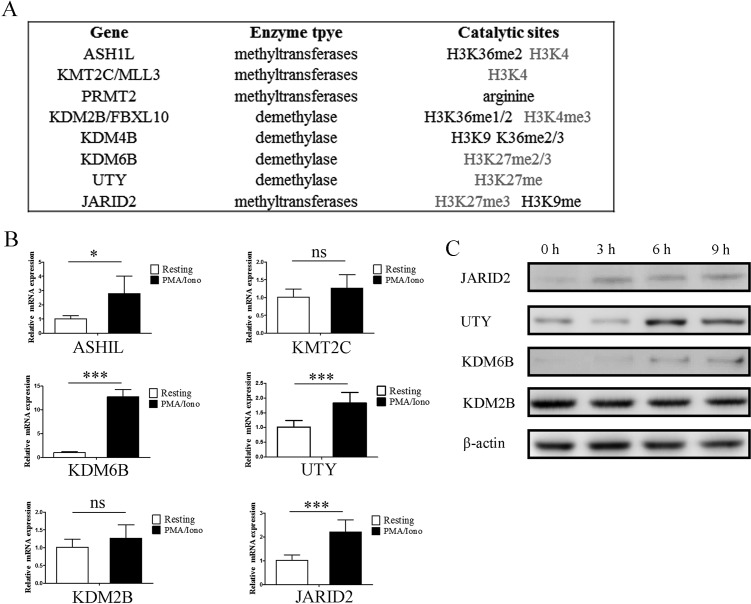
Gene expression of histone methytransferases and demethylases screened from microarray results were verified by qPCR and western blot **A**. The substrate specificities of the indicated enzymes. **B**. Verification of the microarray results by qPCR analysis of the indicated genes in NK92MI cells. Results are represented as fold change over control with glyceraldehyde 3-phosphate dehydrogenase (GAPDH) as a negative control. Error bars represent mean ± SD for three independent experiments (*p < 0.05, ***p < 0.01, and two-tailed unpaired t test). NK92MI cells were stimulated with PMA plus Ionomycin for 6 h. **C**. Verification of the microarray results by western blot of the indicated genes in NK92MI cells. NK92MI cells were stimulated with PMA plus Ionomycin for indicated time points.

Eight histone methytransferases and demethylases were screened out and further analysis was performed in detail. Interestingly, we noticed that 75% of these enzymes are associated with H3K4 methylation and H3K27 methylation (Figure 2A). Microarray results of the indicated genes were confirmed by qPCR analysis in NK92MI cells (Figure 2B). ASHIL, KDM6B, UTY and JARID2 were upregulated following stimulation with PMA/Iono, KDM6B upregulated more than 12 fold. The upregulation of KDM6B, UTY and JARID2 was also confirmed by western blot (Figure 2C). ASHIL is a histone methyltransferase that specifically methylates Lys-4 of histone H3, whereas KDM6B and UTY are histone demethylases that specifically demethylates Lys-27 of histone H3. In that both upregulation of H3K4 modification and downregulation of H3K27 modification are associated with transcriptional activation, it is reasonable to believe that the upregulation of these methytransferase and demethylase genes plays a critical role for enhancing the expression of genes which are tightly regulated by histone modification. However, there is no obvious difference of global modification by H3K27me3, H3K4me3, H3K9me2 and H3K36me3 (Supplementary Figure 2). This implies that the induced expression of indicated methytransferases and demethylases may only impact limited gene loci instead of the global modification state.

### Identifying the relationship between histone modification states and gene expression levels in resting NK cells

**Figure 3:**
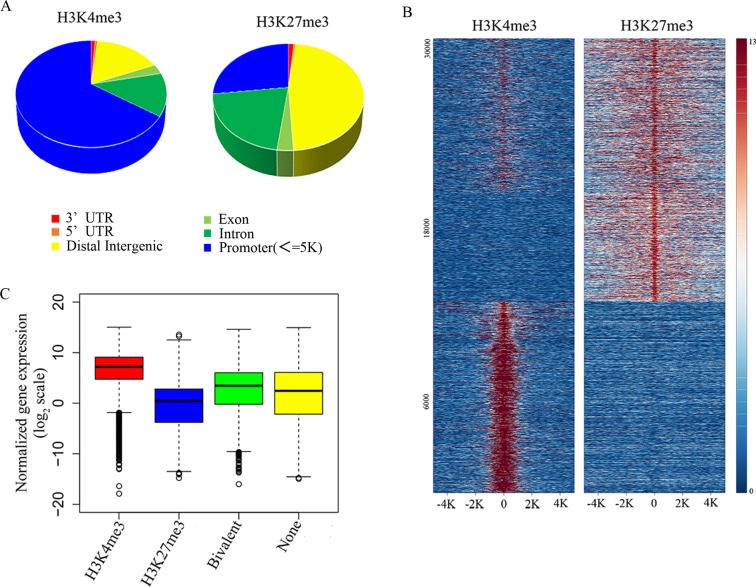
Identifying the relationship between histone modification states and gene expression levels in resting NK cells **A**. Genomic distribution of H3K4me3 and H3K27me3 modifications determined by ChIP-seq analysis. **B**. ChIP-seq density heatmap of H3K4me3 and H3K27me3 on their 30320 binding sites (y-axis) in a 5-kb window centered on gene transcription start sites. **C**. The box plot of normalized gene expression values in the microarray.

In order to explore the functional significance of H3K4me3 and H3K27me3 modification in NK cells during target cell recognition, we analyzed the genome-wide modification targets of the H3K4me3 and H3K27me3 by chromatin IP-based deep sequencing (ChIP-seq). With a p value cutoff of 10^−5^, we identified 22370 H3K4me3-specific modification sites, and 39880 H3K27me3-specific modification sites in resting NK cells. H3K4me3 modification sites were enriched at gene promoters, while H3K27me3-specific binding sites were localized broadly across the genome (Figure 3A). K-means clustering of peaks at RefSeq gene promoters into three clusters showed that genes enriched three groups including all bivalently marked gene groups, monovalently H3K4me3-marked gene groups, and monovalently H3K27me3-marked gene groups (Figure 3B).

To understand the relationship between gene expression pattern and each type of modification, the data from H3K4me3 and H3K27me3 groups were then analyzed to find the overlapping DNA sequences that localized to gene promoters, and these promoters were considered to be the targets of histone modification. The corresponding genes to these promoters were then overlapped with the microarray data, and we found that the H3K4me3 marked gene groups displayed a high gene expression level, whereas H3K27me3 marked gene groups displayed a low gene expression level (Figure 3C). At the same time, all bivalent marked gene groups indicated low or non-expressing genes (Figure 3C). Therefore, the histone modification states are significantly correlated with gene expression levels.

### Dynamic chromatin states were crucial for NK cell activation

Poised or bivalent chromatin - chromatin domains modified with both the activation-associated histone mark H3K4me3 and the repression-associated mark H3K27me3, were first identified at the developmental gene promoters region in embryonic stem cells (ESCs) [12], and recently shown to have the capacity to influence biological processes in the context of differential and cellular responses to environmental cues.

**Figure 4:**
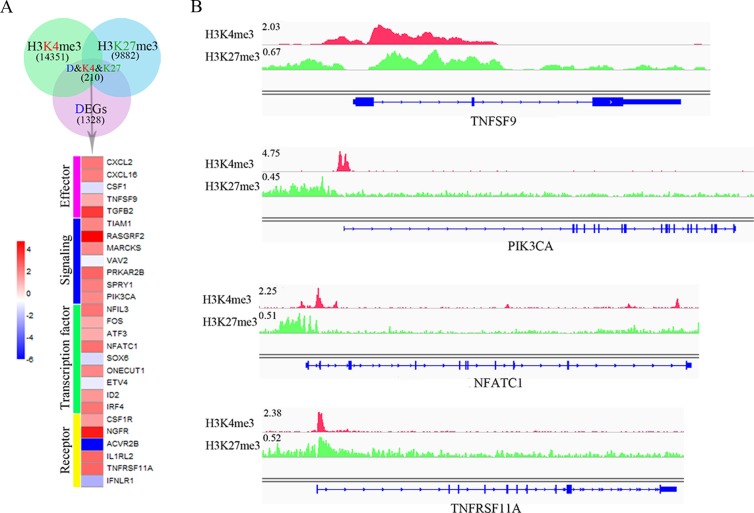
Dynamic chromatin states may be crucial for NK cell activation **A**. Venn diagram shows the overlap of 1328 DEGs (pink), 9882 genes targeted by H3K27me3 (blue) and 14351 genes targeted by H3K4me3 (green). **B**. The modification of H3K4me3 and H3K27me3 on representative target genes, TNFSF9, PIK3CA, NFATC1 and TNFRSF11A. The height of the profile indicates the number of sequenced reads, shown in y-axis.

In general, poised genes show a low or non-expressing level and are shifted rapidly above their baseline expression level after responses to environmental cues. To further investigate the bivalent modification state and NK cell function, 210 DEGs that carry both H3K4me3 and H3K27me3 modifications were found. Then, we manually identified 27 genes that are associated with immune functions, which encode effectors, signaling molecules, transcription factors and receptors (Figure 4A). Interestingly, most of these genes (77.8%) were upregulated (Figure 4A) during target recognition process, and bivalent modification peaks localized to the proximal promoter of the target genes (Figure 4B). These data imply that the bivalent chromatin state may play a key role during NK cell activation.

### Genetic program may be directly altered by dynamic chromatin states during activation

**Figure 5:**
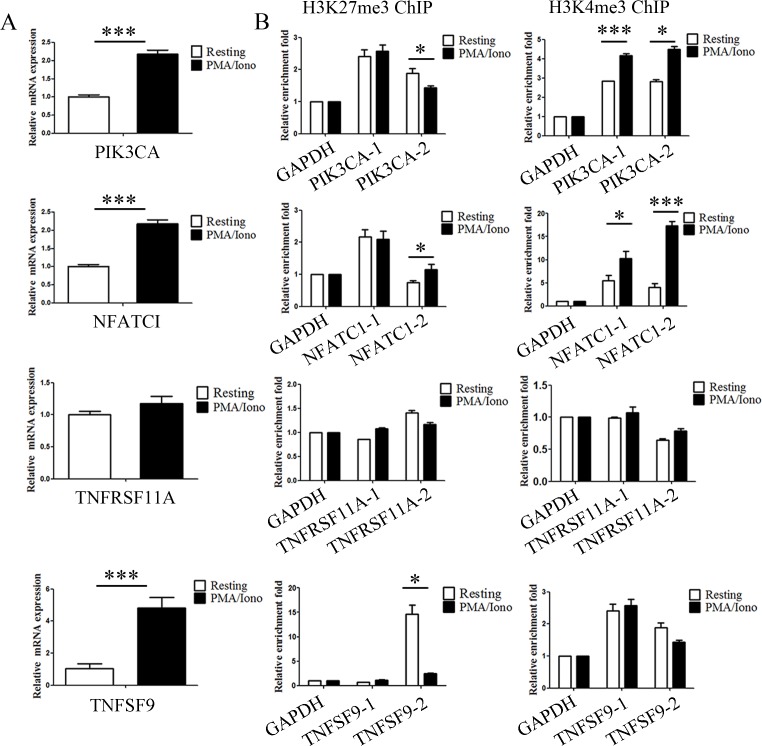
Genetic program may be directly altered by dynamic chromatin states during activation **A**. Verification of the microarray results by qPCR analysis of the indicated genes in NK92MI cells. NK92MI cells were stimulated with PMA plus Ionomycin for 6 h. **B**. Verification of the ChIP-seq results by qChIP analysis of the indicated genes in NK92MI cells. Results are represented as fold change over control with GAPDH as a negative control. Error bars represent mean ± SD for three independent experiments (*p < 0.05, ***p < 0.01, and two-tailed unpaired t test).

Activation of NK cells induced the expression of several histone methyltransferases and demethylases that relate to dynamic bivalent modification and transcriptional activation. In addition, we noticed that the expression of some genes critical for immune functions were upregulated in our microarray analysis. In light of the previous data, we hypothesized that these upregulated genes are the result of dynamic bivalent modification. In our experiments, quantitative PCR (qPCR) analysis was first performed to detect the expression level of PIK3CA, NFATC1, TNFRSF11A and TNFSF9, and the increased expression levels of PIK3CA, NFATC1 and TNFSF9 were verified to be consistent with the microarray results (Figure 5A). Then, to validate the ChIP-seq results, quantitative ChIP (qChIP) analysis in NK92MI cells using specific antibodies against H3K27me3 and H3K4me3 on selected genes including PIK3CA, NFATC1, TNFRSF11A and TNFSF9 was carried out, and showed a strong enrichment of H3K27me3 and H3K4me3 on the promoters, especially for H3K4me3 modification on the NFATC1 promotor and H3K27me3 modification on the TNFSF9 promotor (Figure 5B). Activation of NK cells resulted in a reduction of the H3K27me3 levels at the promoters of PIK3CA and TNFSF9 and a marked increase of the H3K4me3 levels at the promoters of PIK3CA and NFATC1 (Figure 5C). These data indicate that NK cell activation altered bivalent modification resulting in the increased expression of genes associated with immune activation.

### NK cell degranulation and cytokine production were different under several chromatin states

**Figure 6:**
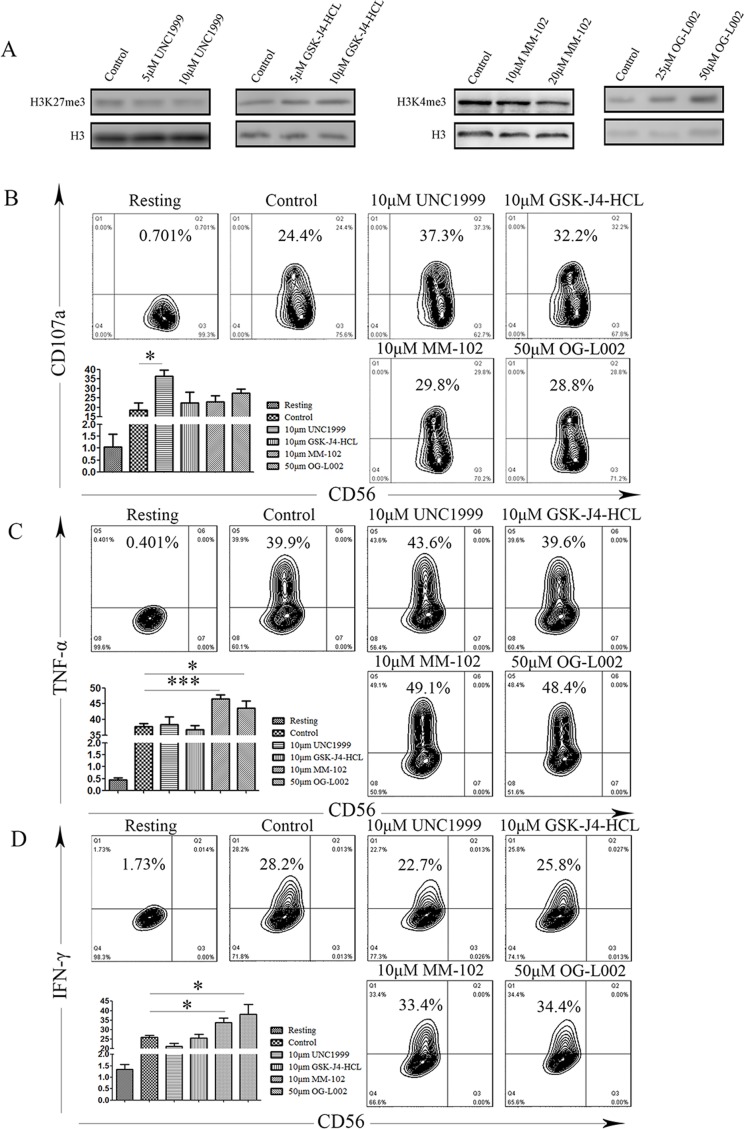
Difference of NK cell degranulation and cytokine production under several chromatin states **A**. Representative western blot of the indicated histone modifications. In NK92MI cells after treatment with DMSO (control), UNC1999, GSK-J4-HCL, MM102 or OG-L002 for 48h (same set-up throughout). **B**. NK cells were gated on forward scatter/side scatter plots, and the profiles show CD56 *versus* CD107a mAb staining. Gates indicate the percentage of CD107a positive NK cells. The percentage of CD107a positive is presented as the mean of 3 independent experiments. Bars indicate SD. **C**. After stimulation, NK92MI cells were surface stained with fluorochrome-conjugated anti-CD56 mAb, fixed, permeabilized, and stained intracellularly with fluorochrome-conjugated mAbs to IFN-γ and TNF-α **D**.. The percentage of NK cells producing IFN-γ and TNF-α, as indicated, was determined by flow cytometry. Values represent mean mean±SD of at least 3 independent experiments. (*p < 0.05, ***p < 0.01, and two-tailed unpaired *t* test).

Subsequent to activation, following the integration of complex signals, NK cells rapidly release cytotoxic granules containing perforin and granzyme and also secrete proinflammatory cytokine IFN-γ and TNF-α, which are important for innate and adaptive immunity against intracellular infections and for tumor control. To further investigate that dynamic chromatin states are crucial for NK cell activation, we used a series of small-molecule compounds that specifically target H3K4 and H3K27 methyltransferases or demethylases, including UNC1999, GSK-J4-HCL, OG-L002, MM102, in order to modify the chromatin state of the NK92MI cell line. We analyzed their immunological function after stimulation with PMA and Ionomycin, using degranulation and secretion of IFN-γ and TNF-α as indicators for NK cell activation. We verified the biochemical function of these small-molecule compounds by western blot, and found that these compound-mediated functions are concentration-dependent (Figure 6A). We measured degranulation based on cell-surface expression of the lysosomal protein CD107a (LAMP-1), using flow cytometry. Resting NK cells did not stain with anti-CD107a mAb. When NK cells were stimulated with PMA and Ionomycin, CD107a expression was detected on 24.4% of the NK cells (Figure 6B). Notably, degranulation was induced in the UNC1999 group compared to the control group (18.67% *vs* 36.53%, *P* = 0.01; Figure 6B). In contrast, other groups did not significantly induce surface expression of CD107a (Figure 6B). To evaluate if methyltransferase or demethylase could affect cytokine production of individual cells, multicolor flow cytometry was performed. We observed increased expression of INF-γ was after treatment with OG-L002 and MM102 (OG-L002: 25.96% *vs* 33.66%, *P* = 0.0195; MM102: 25.95% *vs* 38.22, *P* = 0.042; Figure 6C). In a similar manner, the expression of TNF-α was also up-regulated after treatment with OG-L002 and MM102 (OG-L002: 37.78% *vs* 46.48%, *P* = 0.0009; MM102: 37.78% *vs* 43.62, *P* = 0.0438; Figure 6D).

Together, these data provide insights into the dynamic chromatin states of NK cell activation, illustrating that several small-molecule inhibitors may alter the NK cells’ cytotoxicity function by affecting their chromatin state.

## DISCUSSION

Histone modification is crucial in the regulation of all DNA-based processes, such as transcription. Covalent modification of histones by chromatin-modifying enzymes can fundamentally alter the organization and function of chromatin, and serves as a crucial regulator of cell fate determination, playing an essential role in many biological processes, including immune regulation [13–16]. According to our recent data, inhibition of histone methyltransferase Ezh2 enzymatic activity through small molecule inhibitors could enhance NK cell lineage commitment and promote the survival of both NKp and its progeny. This result is confirmed by the EZH2 conditional knockout mice [17]. In addition to the developmental process, Bluestone and his colleagues found that Ezh2 bridges cellular activation and the maintenance of mature Treg (close relative to NK cell) identity by responding to CD28 signals and supporting the Foxp3-driven gene-expression program [18]. While the role of receptors, signaling components, calcium influx, immune synapse, cytokines, and cytolytic granules in NK cytotoxicity during activation is established, the role of chromatin-modifying enzymes in NK cytotoxicity of mature, differentiated cells after cellular activation is still less clear. In this study, we comprehensively analyze a set of microarray data and two sets of ChIP-seq data and then explore the internal connection of histone state and NK cell cytotoxicity.

Unlike T cells, NK cells are always switched on and can recognize target cells, “dangerous” cells exhibiting a reduced or altered MHC presentation along with ample expression of the activating ligands. From the initial contact with a target cell to the direct delivery of lytic-granule contents to lyse the target cell, NK cells transform into an entirely different state through several distinct stages. Each of the individual stages in NK cell activation is regulated, and this tight regulation is based on the precise level of gene differential expression. One of the mechanisms that allows eukaryotic cells to tightly regulate gene expression is transcriptional control, especially during NK activation. For example, the IFN-γ gene has multiple binding sites for transcription factors including AP-1, NF-κB, and NFAT, all of which contribute to the activation of the IFN-γ gene transcription in response to receptor and/or cytokine stimulation [19, 20]. Furthermore, the expression of other effectors, for example perforin, granzyme, and TNF-α, was also affected by transcription factors during NK cell activation [20]. Here we show that the expression of IFN-γ and NFATC1 were both upregulated when responding to target cell recognition, and we believe that the upregulated expression of IFN-γ was at least partially due to the upregulated expression of NFATC1. We also identified 23 other transcription factors that may regulate certain effector or signaling components that are important for the progression of activation.

The histone methylation modification state regulates chromatin organization leading to either activation or repression of gene expression. For these reasons, we performed a survey of all differentially expressed histone methyltransferase and demethylase genes upon activation of human NK cells. The results revealed that the expression of many methyltransferase and demethylase genes were affected during target cell recognition, which implies that the histone methylation modification state of NK cells were changed in response to target cell recognition. Furthermore, ChIP-seq analysis identified that several genes exhibited bivalent modification by both H3K4me3 and H3K27me27. Interestingly, most of these genes were upregulated upon activation, suggesting that these genes are under the ‘poised’ state. Furthermore, qChIP analysis in NK92MI cells revealed that activation of NK cells resulted in a reduction of the recruitment of the H3K27me3 modification at the promoters of PIK3CA and TNFSF9 and a marked increase of the H3K4me3 modification at the promoters of PIK3CA and NFATC1. These results imply that histone modification may play an important role during NK cell activation.

Several lines of evidence show that the histone modification state may have a profound impact on the entire activation process. To this end, we conducted a series of studies with small-molecule compounds that specifically targeted of H3K4 and H3K27 methyltransferases or demethylases to further investigate the relevance of histone modification and NK cell cytotoxicity. Interestingly, degranulation was induced in the UNC1999 group relative to the control group. In addition, the expression of INF-γ and TNF-α were upregulated after treatment with OG-L002 and MM102. These results imply that small-molecule inhibitors of methytransferases or demethylases may strengthen NK cellular cytotoxicity and possibly suggesting a new therapeutic strategy in cancer immunotherapy. The contribution of histone modification to NK cell cytotoxicity requires further investigation. Moreover, it remains unknown whether similar mechanisms exist in peripheral blood NK cells.

## MATERIALS AND METHODS

### Cell culture

The immortalized human NK cell line, NK92MI was obtained from the American Type Culture Collection (CRL-2408) and maintained in α-Minimum Essential Medium (Gibco) supplemented with 0.2 mM inositol (Sigma), 0.1 mM 2-mercaptoethanol (Gibco), 0.02 mM folic acid (Sigma), 12.5% fetal serum (Gibco), 12.5% horse serum (Gibco), and plated at 2-3 × 10^5^ viable cells/mL at 37°C and 5% CO_2_ in T-75 culture flasks and passaged every 48h.

### Antibodies and fluorescent reagents

Fluorochrome-conjugated monoclonal antibodies (mAbs) used for flow cytometry were anti-CD56 (BD Biosciences), anti-CD107a (BD Biosciences), anti-TNF-α (BD Biosciences), and anti-IFN-γ (BD Biosciences).

### Intracellular staining of cytokines

NK92MI cells (5×10^5^) were stimulated with PMA and Ionomycin (Sigma) for one hour at 37°C in 5% CO_2_. Brefeldin A (BD Biosciences) was then added to the cultures, which were incubated for five more hours. After six hours of total incubation, the cells were centrifuged and resuspended in 50μL of staining buffer (phosphate-buffered saline supplemented with 2% fetal bovine serum) followed by immunostaining with fluorochrome-conjugated mAbs for surface markers. Cells were washed, fixed with Fixation/Permeabilization Kit (BD Biosciences), and intracellularly stained with fluorochrome-conjugated mAbs to TNF-α and IFN-γ. Finally, cells were washed and analyzed on a Fortessa flow cytometer (BD Biosciences). Data analysis was performed using FlowJo 7.6 software (TreeStar).

### Degranulation assay

NK92MI cells (5×10^5^) were stimulated with PMA and Ionomycin (Sigma) for four hour at 37°C in 5% CO_2_. Then the cells were centrifuged and resuspended in 50μL of staining buffer (phosphate-buffered saline supplemented with 2% fetal bovine serum) added fluorochrome-conjugated mAbs for CD56 and CD107a for 30 min on ice. The cells were washed and analyzed on a Fortessa flow cytometer (BD Biosciences). Data analysis was performed using FlowJo 7.6 software (TreeStar).

### RNA isolation and real-time PCR (qPCR)

Total cellular RNAs were isolated with the TRIzol reagent (Invitrogen) and used for the first strand cDNA synthesis with the Reverse Transcription System (Roche). Quantitation of all gene transcripts was done by qPCR using Power SYBR Green PCR Master Mix and an ABI 7500 fast sequence detection system (Applied Biosystems) with the expression of GAPDH as the internal control. Primers are provided in Supplementary Table 1.

### Western blot

Cell lysates were resolved using 12% SDS-PAGE gels and transferred onto polyvinylidene fluoride membranes. For western blot analysis, membranes were treated with appropriate antibodies and incubated overnight at 4°C followed by incubation with a secondary antibody at room temperature.

### Chromatin immunoprecipitation

Cells were cross-linked with 1% formaldehyde at room temperature for 10 min and stopped with 1.25 M Glycine. Cells were then rinsed with ice-cold PBS twice and suspended in Lysis buffer (50 mM Tris·HCl, pH8.0, 10 mM EDTA, 1% SDS, 1 mM PMSF, 1× Protease Inhibitor cocktail) and incubated for 10 min on ice. Cells were sonicated (Bioruptor) and subsequently centrifuged for 10 min. Supernatants were collected and diluted in Dilution buffer (20mMTris·HCl, pH8.0, 2mMEDTA, 150mM NaCl, 1% Triton X-100, 1× Protease Inhibitor Mixture). Immunoprecipitation was performed overnight at 4°C with specific antibodies followed by the addition of Dynabeads-protein G (Life Technologies) for an additional 2 h of incubation. Precipitates were washed sequentially for 10 min each in low salt buffer (20 mM Tris·HCl, pH 8.0, 0.1% SDS, 1% Triton X-100, 2 mM EDTA, 150 mM NaCl), high salt buffer (20 mM Tris·HCl, pH 8.0, 0.1% SDS, 1% Triton X-100, 2 mM EDTA, 500 mM NaCl), LiCl buffer (10 mM Tris·HCl, pH 8.0, 0.25 M LiCl, 1% Nonidet P-40, 1% deoxycholate, 1 mM EDTA). Precipitates were then washed three times with TE buffer and extracted with Elution buffer (20 mM Tris·HCl, pH 7.5, 5 mM EDTA, 50 mM NaCl, 1% SDS) and heated at 65°C for at least 2 h to reverse the cross-linking. DNA fragments were purified with a PCR Purification kit (Qiagen). Primers are provided in Supplementary Table 1.

### Differential expression analysis between CD107 positive and negative samples

Gene expression profiles of CD107 positive and negative NK cells from human peripheral blood which that were co-cultured with tumor target cell line K562 for 4 hours with brefeldin followed by immunostaining for granule exocytosis marker CD107, were obtained from Gene Expression Omnibus (GEO) with the accession number of GSE55977, deposited by Vaz et al. Through PreProcessCore package of R, the raw data was normalized and differential expression genes (DEGs) between CD107pos and CD107neg pooled samples were screened out based on limma package with the criteria of |log FC| > 1 and *P* value < 0.05. The significantly enriched (*P* value < 0.05) KEGG pathways of DEGs were identified by the Database for Annotation, Visualization and Integrated Discovery (DAVID, https://david.ncifcrf.gov/) [4] and the Kyoto Encyclopedia of Genes and Genomes (KEGG) pathways were visualized and clustered through the GOplot package [5].

### Genome wide binding profiles analysis of H3K4me3 and H3K27me3

To explore the genome wide modification profiles of H3K4me3 and H3K27me3, Chromatin Immunoprecipitation combined with next generation sequencing (ChIP-Seq) datasets GSM1027301 (H3K4me3) and GSM1027291 (H3K27me3) were downloaded from the Gene Expression Omnibus (GEO, http://www.ncbi.nlm.nih.gov/geo/). The raw fastq files were preprocessed *via* removing the reads containing more than 5 bases with a quality score < 20, and Bowtie 2, an ultrafast and memory-efficient aligning tool, was used for the mapping of the remaining reads to the UCSC GRCh37/hg19 genome with maximum of 2 mismatches. Through the Model-based Analysis for ChIP-Seq (MACS) version 2, developed by Liu et al [6], the H3K4me3 and H3K27me3 specific binding sites (also known as peaks) were identified with the thresholds of *P*value < 10^−5^. Peaks were annotated with several features, including their nearby genes, distance to the nearest transcription start sites (TSS), and location features (such as promoter, intron, exon, etc.) using ChIPseeker [7], an R Bioconductor package.

### Statistics

Statistical analysis was performed using two-tailed unpaired t test. P values of less than 0.05 were considered significant.

## SUPPLEMENTARY MATERIALS FIGURES AND TABLES



## Abbreviations

NK cell: Natural Killer cell
ChIP-seq: Chromatin Immunoprecipitation-Sequencing
PMA: Phorbol Myristate Acetate
MTOC: Microtubule Organizing Center
CaM: Ca^2+^ Sensor Calmodulin
PTMs: Post-Translational Modifications
GEO: Gene Expression Omnibus
DEGs: Differential Expression Genes
MACS: Model-based Analysis for ChIP-Seq
TSS: Transcription Start Sites
DAVID: Database for Annotation, Visualization and Integrated Discovery
KEGG: Kyoto Encyclopedia of Genes and Genomes
ESCs: Embryonic Stem Cells
qChIP: quantitative Chromatin Immunoprecipitation
PMA: Phorbol Myristate Acetate
Iono: Ionomycin.
