# Supplementary material for: Chromatin state dynamics during NK cell activation
Source: Oncotarget. 2017 Mar 29;8(26):41854–65. doi: 10.18632/oncotarget.16688 (PMC5522033; doi:10.18632/oncotarget.16688)
Supplement: Supplementary file 1 [file oncotarget-08-41854-s001.pdf]

Chromatin state dynamics during NK cell activation

Supplementary Material

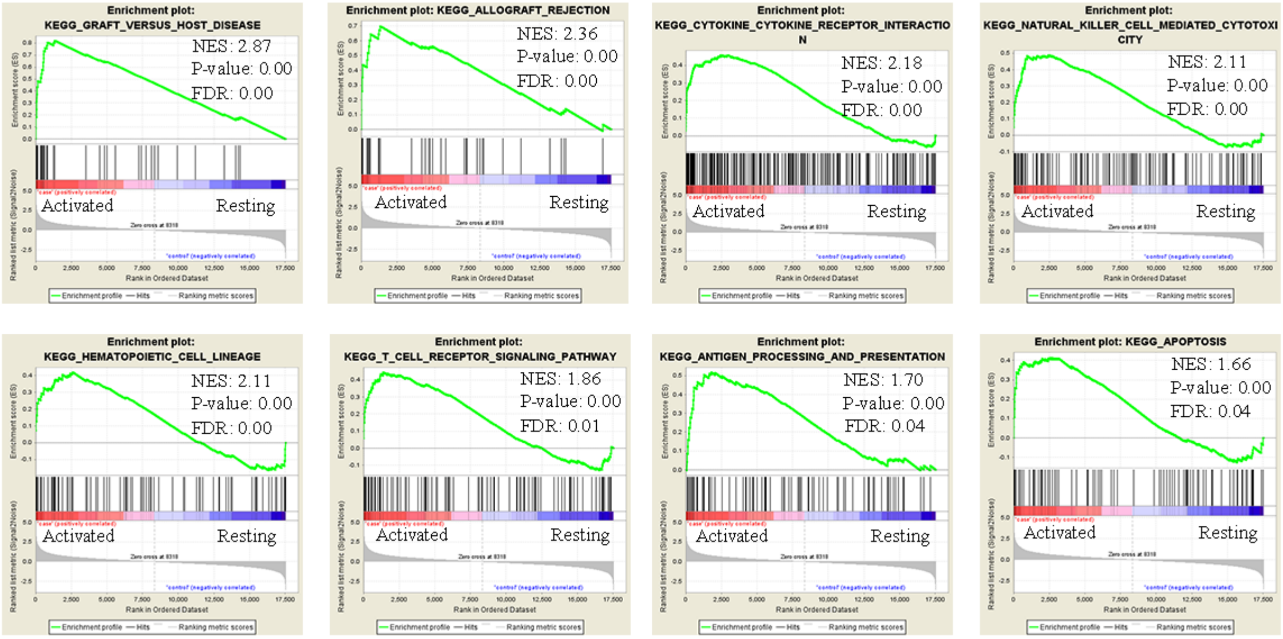

Supplementary Figure 1: Unbiased GSEA enrichment plot of indicated gene sets in expression data from activating NK cell compared to resting NK cell.

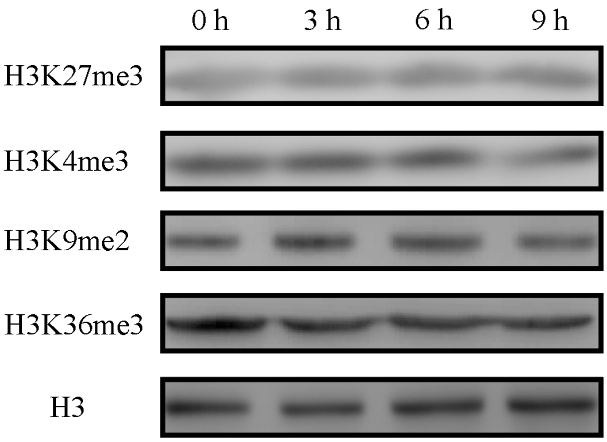

Supplementary Figure 2: Representative western blots were performed with antibody against H3K27me3, H3K4me3, H3K9me2, H3K36me3 and H3 as control. NK92MI cells were stimulated with PMA plus Ionomycin for indicated time points.

Supplementary Table 1: RT-qPCR primers

Genes	Primers
PIK3CA-F	CCACGACCATCATCAGGTGAA
PIK3CA-R	CCTCACGGAGGCATTCTAAAGT
TNFRSF11A-F	CACCAAATGAACCCCATGTTTAC
TNFRSF11A-R	GGACTCCTTATCTCCACTTAGGC
NFATC1-F	CACCGCATCACAGGGAAGAC
NFATC1-R	GCACAGTCAATGACGGCTC
TNFSF9-F	CTCCGTTTCACTTGCGCTG
TNFSF9-R	TCAGTGTGAAGATGGACGCC
ASH1L-f	ACACTGTCCTTCAAAACGAGAC
ASH1L-r	GAAGAGTAGATGGCGTTGCATTA
KMT2C-f	TGGGTTCACCTAGAGTGTGAC
KMT2C-r	CTGGCTGTAAACGATCCATCTC
KDM2B-f	TGTCCCAGTTTGTGCGTTACT
KDM2B-r	GGACGCTTGACCAAGTGCT
KDM6B-f	CACCCCAGCAAACCATATTATGC
KDM6B-r	CACACAGCCATGCAGGGATT
UTY-f	CACAAAGAAGTTGCTCAGGTACG
UTY-r	TGTGGTTGTCGATTAGAGACAGA
JARID2-f	ACCAGTCTAAGGGATTAGGACC
JARID2-r	TGCTGGGACTATTCGGCTGA

Chip-qPCR primers

Genes	Primers
PIK3CA-CHIP-1F	TCAAGGAGGAACCGATGCTG
PIK3CA-CHIP-1R	TAAGGGTTCGGTACTGTGCG
PIK3CA-CHIP-2F	AGCTTTGCCAGAATCCTCCC
PIK3CA-CHIP-2R	GCCATGCCTTGCCTAGAGTC
TNFRSF11A-CHIP-1F	ATGATTCCCTCCGTCCCTGA
TNFRSF11A-CHIP-1R	GAGGGTCAAGTCCAGCATCC
TNFRSF11A-CHIP-1F	TGAAAGGGCCAATGTGGTGA
TNFRSF11A-CHIP-1R	GCCAGCAGCCACTATCTCTT
NFATC1-CHIP-1F	CTAGTCTCCAGACCCTGGCT
NFATC1-CHIP-1R	CGGTGGTCACTTGAGAGCTT
NFATC1-CHIP-2F	AAACCTACGAGGAGGCAGGA
NFATC1-CHIP-2R	CCTCTGTTTAATGCCGTTGCC
TNFSF9-CHIP-1F	CCCGCTACGAGACCCTATCT
TNFSF9-CHIP-1R	CTGAGGGGTTGAGGACTGTG
TNFSF9-CHIP-2F	AGGAGACCCCCAGTTCCTTT
TNFSF9-CHIP-2R	TCTTCTTTCCAGCCCCCTTG
NFATC1-CHIP-1F	CTAGTCTCCAGACCCTGGCT
NFATC1-CHIP-1R	CGGTGGTCACTTGAGAGCTT
NFATC1-CHIP-2F	AAACCTACGAGGAGGCAGGA
NFATC1-CHIP-2R	CCTCTGTTTAATGCCGTTGCC

TNFSF9-CHIP-1F	CCCGCTACGAGACCCTATCT
TNFSF9-CHIP-1R	CTGAGGGGTTGAGGACTGTG
TNFSF9-CHIP-2F	AGGAGACCCCCAGTTCCTTT
TNFSF9-CHIP-2R	TCTTCTTTCCAGCCCCCTTG
